# FGF/FGFR signaling at the immune-stromal interface in autoimmune rheumatic diseases: from tissue remodeling to translational opportunities

**DOI:** 10.3389/fimmu.2026.1875220

**Published:** 2026-07-22

**Authors:** Sheng Zhong, Xiaohui Meng, Zheng Xiang, Guowei Qiu, Songtao Sun, Jun Xie, Lianbo Xiao

**Affiliations:** 1 Department of Orthopedics, Guanghua Hospital Affiliated to Shanghai University of Traditional Chinese Medicine, Shanghai, China; 2 Department of Orthopedics and Rheumatology, Wuxi Affiliated Hospital of Nanjing University of Chinese Medicine, Wuxi, China

**Keywords:** FGFR, fibroblast growth factor, immune-stromal interface, lupus nephritis, rheumatoid arthritis, Sjögren’s disease, spondyloarthritis, systemic sclerosis

## Abstract

The fibroblast growth factor (FGF)/FGF receptor (FGFR) signaling system regulates tissue repair, angiogenesis, extracellular matrix remodeling, fibrosis, and bone metabolism. Although FGF/FGFR biology has been extensively studied in oncology, development, and fibrotic lung disease, its relevance to systemic autoimmune rheumatic diseases remains scattered across disease-specific and unevenly supported literatures. This narrative, evidence-informed review synthesizes current evidence on FGF/FGFR signaling in rheumatoid arthritis (RA), systemic lupus erythematosus/lupus nephritis (SLE/LN), systemic sclerosis (SSc), spondyloarthritis/psoriatic arthritis (SpA/PsA), and Sjögren’s disease. We explicitly distinguish mechanistically supported axes from biomarker-level associations and extrapolated regenerative concepts. In RA, FGF10/FGFR1 and FGF2/FGFR3 have the strongest tissue-level support, linking relapse-prone synovial fibroblast niches, fibroblast-like synoviocyte activation, angiogenesis, and bone erosion. In SLE/LN, FGF23/Klotho is best interpreted as an emerging renal-metabolic and inflammatory biomarker axis rather than a validated therapeutic target, although reported associations with urinary MCP-1/CCL2 suggest testable links to renal chemokine-mediated inflammation. In SSc-ILD, nintedanib demonstrates that FGFR-containing multi-kinase networks are clinically tractable, but the FGFR-specific contribution remains unresolved. In SpA/PsA, FGF2-mediated angiogenesis-osteogenesis coupling and FGF7/FGFR2IIIb signaling provide candidate mechanisms for entheseal remodeling, whereas direct human entheseal validation is still lacking. In Sjögren’s disease, FGF7/FGF10/FGFR2b signaling supports a regenerative hypothesis derived mainly from salivary gland developmental biology and organoid studies. We propose an axis-prioritization translational framework that specifies ligand-receptor pairs, patient subgroups, validation tissues, delivery strategies, pharmacodynamic endpoints, and clinical or imaging readouts. This review is not a systematic review or meta-analysis; rather, it provides a structured roadmap for tissue-level validation, longitudinal biomarker studies, and short-course proof-of-mechanism trials before clinical translation.

## 1 Introduction

Systemic autoimmune rheumatic diseases encompass a heterogeneous group of disorders—including rheumatoid arthritis (RA), systemic lupus erythematosus (SLE), systemic sclerosis (SSc), spondyloarthritis (SpA), psoriatic arthritis (PsA), and Sjögren’s disease—that share immune dysregulation but differ markedly in the tissues in which irreversible damage occurs (1). Despite major therapeutic advances targeting TNF, IL-6, B cells, T-cell co-stimulation, JAK-STAT signaling, and type I interferon pathways, many patients continue to develop joint erosion, fibrosis, entheseal new bone formation, renal or vascular damage, or glandular dysfunction (2). This dissociation between systemic inflammatory control and tissue damage has shifted attention toward stromal, endothelial, epithelial, and osteochondral compartments as active determinants of disease persistence and structural progression (3).

At these disease sites, tissue-resident fibroblasts, myofibroblasts, endothelial cells, epithelial cells, osteoblast-lineage cells, osteoclast precursors, and infiltrating immune cells form self-sustaining pathogenic niches. These niches integrate cytokine exposure, mechanical stress, hypoxia, extracellular matrix cues, and growth factor signaling (3). The immune-stromal interface is therefore not a single cell-cell interaction, but a dynamic tissue program that determines whether inflammation resolves with repair or progresses toward fibrosis, erosion, ossification, or glandular failure.

The fibroblast growth factor (FGF) family comprises 22 members in humans, signaling through four tyrosine kinase receptors (FGFR1-4) and co-receptors including α-Klotho and β-Klotho (4). FGF/FGFR pathways are well positioned to regulate processes central to autoimmune tissue pathology, including fibroblast proliferation, endothelial activation, angiogenesis, matrix remodeling, bone formation and resorption, epithelial repair, and metabolic stress responses (5). Unlike classical pro-inflammatory cytokines such as TNF or IL-6, FGF signaling operates primarily at the interface between immune cells and tissue-resident stromal cells, positioning it as a potential mediator of the critical transition from inflammation to structural remodeling (6). The fact that FGF signaling can be both protective (tissue repair, homeostasis) and pathological (fibrosis, aberrant bone formation, tumor-like FLS behavior) depending on context makes it a particularly nuanced target that demands careful disease-specific analysis.

Previous reviews have addressed FGF biology in cancer, development, idiopathic pulmonary fibrosis, or selected rheumatic-disease contexts (7, 8). However, the autoimmune rheumatic disease literature remains fragmented in two ways. First, evidence strength differs sharply across diseases: RA and SSc-ILD contain tissue-level or clinical translational data, whereas SLE/LN, SpA/PsA, and Sjögren’s disease are supported mainly by biomarker, preclinical, or extrapolated regenerative evidence (9–12). Second, the translational implications of each axis differ: some axes justify tissue-level validation, others only longitudinal biomarker testing, and only a small subset is currently suitable for short-course proof-of-mechanism intervention. A cross-disease synthesis is therefore useful only if it does more than catalogue expression changes; it must rank axes by mechanistic depth and translational readiness.

Several factors make such a synthesis particularly timely. First, recent single-cell and spatial transcriptomic studies have revealed previously unappreciated heterogeneity in synovial and tissue fibroblast populations, including FGF-responsive subsets such as CD55^+^ lining FLS that drive RA relapse (9). These technologies now enable the mapping of FGF-FGFR ligand–receptor interactions at single-cell resolution within disease tissues. Second, the approval of nintedanib for SSc-ILD shows that multi-kinase pathways including FGFR-related signaling can be clinically targeted in autoimmune-associated fibrosis, while leaving the FGFR-specific contribution unresolved (10). Third, the endocrine FGF axis—particularly FGF23/Klotho—has attracted attention as a potential biomarker linking inflammation, mineral metabolism, and organ damage across multiple rheumatic diseases (11, 13), though the clinical significance of these associations remains debated. Fourth, the growing pipeline of selective FGFR inhibitors, FGF ligand modulators (such as burosumab and FGF21 analogues), and local delivery technologies (14) creates a need to evaluate which FGF/FGFR axes represent realistic therapeutic or biomarker opportunities in autoimmunity, as opposed to those with evidence limited to oncology or metabolic disease.

**Figure 1:**
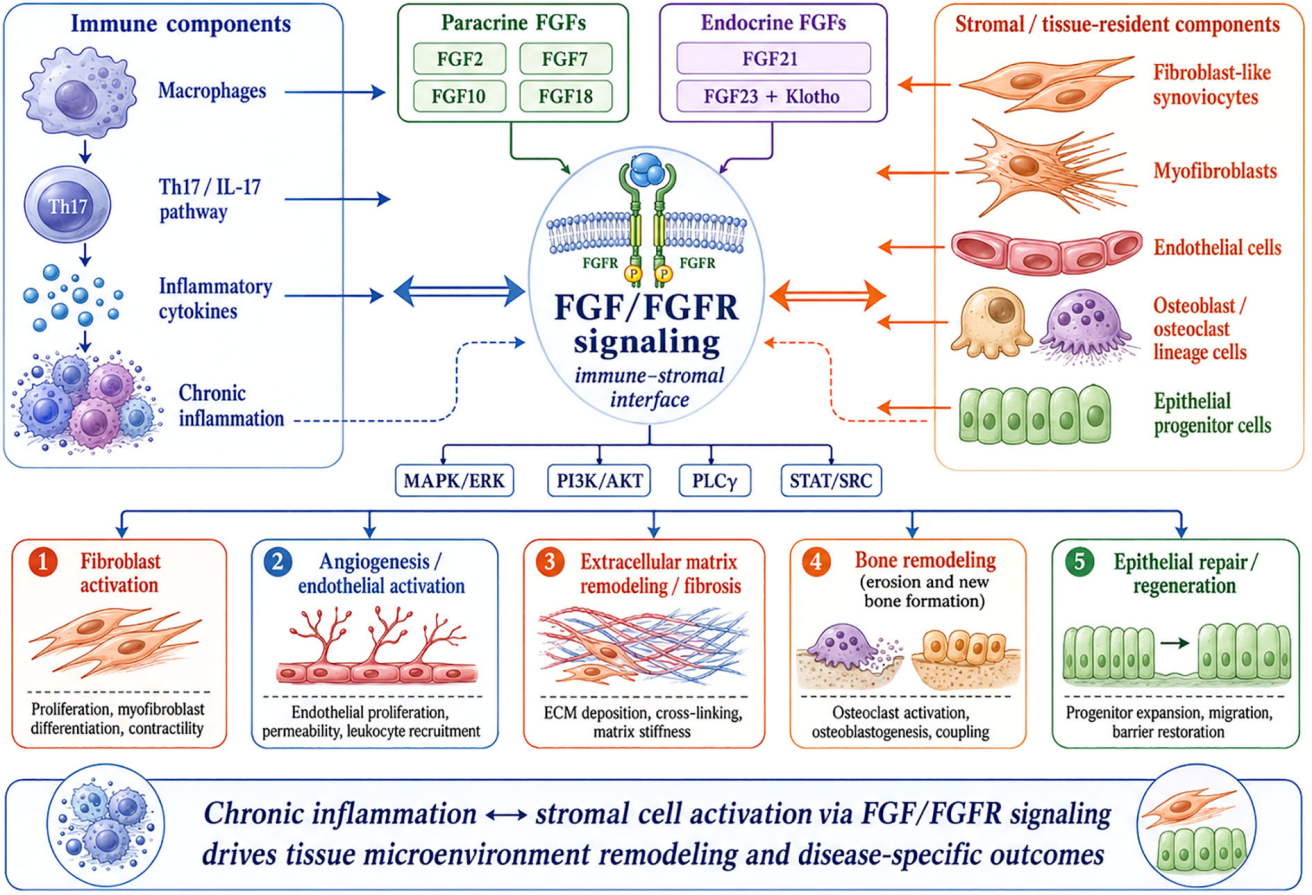
FGF/FGFR signaling at the immune–stromal interface in autoimmune rheumatic diseases. FGF/FGFR signaling links chronic immune activation with tissue-resident stromal and epithelial compartments across autoimmune rheumatic diseases. Paracrine FGFs, including FGF2, FGF7, FGF10, and FGF18, act locally within inflamed synovium, fibrotic tissue, entheses, and exocrine glands to regulate fibroblast activation, angiogenesis, extracellular matrix remodeling, bone remodeling, and epithelial repair. Endocrine FGFs, particularly FGF21 and FGF23, connect inflammation with systemic metabolic, mineral, renal, and vascular pathways through Klotho-dependent signaling. Downstream FGFR activation engages canonical pathways such as MAPK/ERK, PI3K/AKT, PLCγ, and STAT/SRC, with disease-specific outcomes determined by ligand-receptor pairing, target cell type, tissue architecture, inflammatory milieu, and disease stage. This image presents a conceptual framework rather than a uniform mechanism operating equivalently across all diseases.

This review differs from existing FGF reviews in three important ways. Rather than cataloging FGF expression changes across diseases, we organize the evidence around a unifying conceptual framework: FGF/FGFR signaling as an immune-stromal interface that links chronic inflammation with tissue microenvironment remodeling (**Figure 1**). We cover five major autoimmune rheumatic diseases—RA, SLE, SSc, SpA/PsA, and Sjögren’s disease—providing disease-specific mechanistic analysis while explicitly evaluating evidence strength and acknowledging gaps. Finally, we devote substantial attention to translational implications, arguing that FGF-targeted therapy in autoimmune diseases should prioritize tissue-selective, locally delivered, and biomarker-guided approaches rather than systemic pan-FGFR inhibition borrowed from oncology.

In this review, we synthesize current evidence on FGF/FGFR signaling in systemic autoimmune rheumatic diseases, with emphasis on tissue microenvironment remodeling, immune-stromal crosstalk, biomarkers, and translational opportunities.

Because the available evidence varies substantially across diseases, this review intentionally distinguishes mechanistically supported disease contexts from biomarker-level associations and extrapolated regenerative concepts. Accordingly, conclusions regarding SLE, SpA/PsA, and Sjögren’s disease should be interpreted as more hypothesis-generating than those regarding RA and SSc-ILD.

### 1.1 What this review adds

This review does not aim to provide another descriptive catalogue of FGF expression changes in autoimmune rheumatic diseases. Its added value is an evidence-graded, axis-specific translational framework. We ask four questions for each proposed FGF/FGFR axis: (i) Is the evidence derived from diseased human tissue, peripheral biomarkers, animal models, organoids, or non-autoimmune extrapolation? (ii) Is the relevant ligand-receptor pair and FGFR isoform defined? (iii) Which patient subgroup and tissue compartment would be appropriate for validation? (iv) What pharmacodynamic and clinical or imaging endpoints would be needed before therapeutic translation? By applying these questions across RA, SLE/LN, SSc, SpA/PsA, and Sjögren’s disease, we distinguish axes ready for mechanistic validation from those that remain hypothesis-generating.

### 1.2 Narrative literature search strategy and evidence appraisal

This article is a narrative, evidence-informed review rather than a systematic review. We searched PubMed/MEDLINE, Web of Science, and Scopus from inception to May 1, 2026, using combinations of FGF/FGFR-related terms and disease-specific terms for RA, SLE/LN, SSc, SpA/PsA, and Sjögren’s disease. The search was designed to identify representative mechanistic, biomarker, preclinical, clinical, and review literature relevant to FGF/FGFR signaling in autoimmune rheumatic diseases. No PRISMA-based screening process, formal risk-of-bias assessment, protocol registration, or meta-analysis was performed. Therefore, the conclusions should be interpreted as a structured expert synthesis and translational prioritization exercise, not as a quantitative evidence synthesis or definitive ranking of study quality.

The representative PubMed search strategy was: (“fibroblast growth factor” OR FGF OR FGFR OR FGF2 OR FGF10 OR FGF21 OR FGF23 OR Klotho) AND (“rheumatoid arthritis” OR “systemic lupus erythematosus” OR “lupus nephritis” OR “systemic sclerosis” OR “spondyloarthritis” OR “psoriatic arthritis” OR “Sjögren”). The representative PubMed search strategy was adapted for Web of Science and Scopus.

Evidence categories were used not only to describe the literature but also to determine the appropriate translational next step. Axes with strong mechanistic evidence were considered candidates for tissue-level validation and short-course proof-of-mechanism studies. Biomarker-level axes were considered candidates for longitudinal prognostic cohorts, not therapeutic targeting. Preliminary or extrapolated axes were treated as hypothesis-generating and were not presented as established disease mechanisms. This distinction is central to the interpretation of the disease-specific sections and the translational roadmap.

## 2 The FGF/FGFR signaling system: ligands, receptors and downstream pathways

The human FGF superfamily comprises 22 members that can be classified into three functional groups based on their mechanism of action: paracrine FGFs, endocrine FGFs, and intracrine FGF homologous factors (FHFs) (4, 15). Understanding this classification is essential for interpreting the diverse roles of FGF signaling in autoimmune rheumatic diseases, as different FGF subfamilies contribute to distinct pathological processes.

Paracrine FGFs constitute the largest group and include FGF1–10, FGF16–18, FGF20, and FGF22. These ligands bind heparan sulfate proteoglycans (HSPGs) in the extracellular matrix, which restricts their diffusion and confines signaling to local tissue microenvironments (5, 15). Paracrine FGFs of particular relevance to rheumatic diseases include FGF2 (basic FGF/bFGF), a potent inducer of fibroblast proliferation, angiogenesis, and osteoclastogenesis (16); FGF7 (keratinocyte growth factor, KGF), which signals exclusively through the FGFR2IIIb splice variant on epithelial cells (17); and FGF10, which drives branching morphogenesis and tissue repair through FGFR1IIIb and FGFR2IIIb (18).

Endocrine FGFs—FGF19, FGF21, and FGF23—lack the heparan sulfate-binding domain found in paracrine FGFs, enabling them to enter the circulation and act as hormones (19). These endocrine FGFs require obligate co-receptors for signaling: FGF23 signals through FGFR1c in complex with α-Klotho to regulate phosphate homeostasis and vitamin D metabolism (20), while FGF21 signals through FGFR1c/β-Klotho to modulate glucose and lipid metabolism (21). The FGF23/α-Klotho axis has emerged as a biomarker of interest across multiple rheumatic diseases, including RA, SLE, and SSc (11–13).

Intracrine FGF homologous factors (FGF11–14) do not bind FGFRs and instead function intracellularly as regulators of voltage-gated sodium channels (15). These are not further discussed in this review.

FGF signals are transduced through four receptor tyrosine kinases, FGFR1-4, each consisting of an extracellular ligand-binding domain with three immunoglobulin-like loops, a single transmembrane domain, and an intracellular split tyrosine kinase domain (4, 7). Alternative splicing of the third immunoglobulin-like domain in FGFR1–3 generates IIIb and IIIc isoforms with distinct ligand-binding specificities: IIIb isoforms are predominantly expressed on epithelial cells and bind FGF7 and FGF10, while IIIc isoforms are expressed on mesenchymal cells and bind FGF2 and FGF18 (22). This tissue-specific isoform expression creates a directional signaling architecture—epithelial-to-mesenchymal and mesenchymal-to-epithelial—that is directly relevant to immune-stromal interactions in rheumatic diseases.

Upon ligand binding, FGFRs undergo dimerization and transphosphorylation, activating four major downstream cascades (4, 5). The RAS-MAPK/ERK pathway drives cell proliferation and differentiation and is implicated in FLS activation in RA (23). The PI3K-AKT pathway promotes cell survival, migration, and metabolic reprogramming and has been linked to FGF23-mediated IL-1β synthesis in RA (24). The PLCγ pathway generates inositol trisphosphate and diacylglycerol, modulating intracellular calcium and protein kinase C signaling. The STAT/SRC pathway mediates gene transcription responses relevant to inflammation and fibrosis (25). The relative contribution of each downstream cascade varies by FGF-FGFR combination, cell type, and disease context, contributing to the pleiotropic effects of FGF signaling observed across different rheumatic diseases.

FGF signaling is subject to multiple layers of negative regulation that maintain homeostatic balance. Sprouty (SPRY) proteins inhibit RAS-MAPK activation downstream of FGFRs (26). Similar Expression to FGF (SEF/IL17RD) antagonizes FGF-induced ERK phosphorylation. Dual-specificity phosphatases, particularly DUSP6/MKP-3, dephosphorylate and inactivate ERK1/2 (27). Disruption of these negative feedback mechanisms may contribute to sustained FGF signaling in chronically inflamed tissues.

Beyond its canonical roles in development, FGF signaling is increasingly recognized as a regulator of processes central to rheumatic disease pathology. FGF2 is one of the most potent inducers of angiogenesis, promoting endothelial cell proliferation, migration, and tube formation (28). FGF signaling regulates both osteoblast and osteoclast activity, influencing bone formation and resorption (29). In tissue repair and fibrosis, FGFs can exert context-dependent effects—promoting beneficial tissue regeneration or contributing to pathological fibrosis depending on the cellular context, FGF family member, and disease stage (5). This dual nature of FGF signaling—protective versus pathological—is a recurring theme in the disease-specific sections that follow (**Table 1**).

**Table 1:** FGF family members and receptor systems relevant to autoimmune rheumatic diseases.

FGF member	Subfamily	Receptor	Co-receptor	Signaling type	Key biological function	Primary disease relevance
FGF2 (bFGF)	Paracrine (FGF1 subfamily)	FGFR1, FGFR3	HSPG	Paracrine/autocrine	Angiogenesis, fibroblast proliferation, wound repair	RA (FLS activation, bone erosion); SSc (fibrosis); SpA (angiogenesis-osteogenesis)
FGF7 (KGF)	Paracrine (FGF7 subfamily)	FGFR2IIIb	HSPG	Paracrine	Epithelial cell growth, tissue repair	PsA (entheseal ossification); Sjögren (gland repair)
FGF10	Paracrine (FGF7 subfamily)	FGFR1IIIb, FGFR2IIIb	HSPG	Paracrine	Branching morphogenesis, tissue repair	RA (relapse-prone synovial niche); Sjögren (salivary gland regeneration)
FGF18	Paracrine (FGF8 subfamily)	FGFR3	HSPG	Paracrine	Chondrogenesis, cartilage homeostasis	OA/RA (cartilage repair potential)
FGF21	Endocrine (FGF19 subfamily)	FGFR1c	β-Klotho	Endocrine	Metabolic regulation, glucose/lipid homeostasis	SSc (metabolic-immune crosstalk); potential systemic effects
FGF23	Endocrine (FGF19 subfamily)	FGFR1c	α-Klotho	Endocrine	Phosphate homeostasis, vitamin D metabolism	SLE/LN (renal damage biomarker); RA (comorbidity marker); SSc (vascular injury); SpA (bone metabolism)
FGFR1	—	FGF1/2/10 and others	—	—	Widely expressed; mesenchymal signaling	RA (FGF10/FGFR1 synovial relapse axis)
FGFR2 (IIIb/IIIc)	—	FGF7/10 (IIIb); FGF2 (IIIc)	—	—	Epithelial (IIIb) vs mesenchymal (IIIc)	PsA (FGFR2IIIb in enthesis); Sjögren (gland development)
FGFR3	—	FGF2/18 and others	—	—	Chondrocyte, skeletal regulation	RA (FGFR3-RSK2-MAPK in FLS)
α-Klotho	—	—	FGF23 co-receptor	—	Anti-aging, phosphate regulation	SLE (Klotho deficiency in LN); RA/SSc (vascular marker)

## 3 FGF/FGFR signaling at the immune-stromal interface

The concept of the immune-stromal interface has emerged as a central paradigm in rheumatic disease biology, recognizing that tissue-resident mesenchymal cells are not merely structural scaffolds but active participants in disease perpetuation (2, 3). FGF/FGFR signaling occupies a unique position at this interface, serving as a bidirectional communication system between infiltrating immune cells and tissue-resident stromal populations. This section outlines the key mechanisms through which FGF signaling mediates immune-stromal crosstalk, providing the conceptual framework for the disease-specific analyses that follow (**Figure 1**).

### 3.1 Fibroblast activation and pathological phenotype acquisition

Tissue-resident fibroblasts are among the primary cellular targets of FGF signaling. In the context of chronic inflammation, FGF2 and FGF10 can drive fibroblast proliferation, migration, and phenotypic transition toward aggressive, inflammation-sustaining phenotypes (6). In the RA synovium, FLS exposed to FGF2 upregulate matrix metalloproteinases (MMPs), proliferative markers, and pro-inflammatory mediators (16, 23). FGF10/FGFR1 signaling has been shown to maintain a relapse-prone lining FLS population characterized by CD55 expression, suggesting that FGF signals may contribute to the persistence of pathological fibroblast niches even after clinical remission (9). In SSc, fibroblast activation by FGF signaling contributes to excessive extracellular matrix deposition and tissue fibrosis, though the net effect is complicated by evidence that FGF2 may also exert anti-fibrotic properties in certain contexts (30). This context-dependent duality underscores the need for careful analysis of FGF effects at the level of specific ligand–receptor pairs, cell types, and disease stages.

### 3.2 Interactions with macrophages and the Th17/IL-17 axis

FGF signaling does not merely act on stromal cells—it feeds back into the immune system. FGF23 facilitates IL-1β synthesis in RA through PI3K/AKT/NF-κB activation, establishing a direct molecular link between the endocrine FGF axis and inflammasome-related innate immunity (24). FGF21 exerts the opposite effect: in collagen-induced arthritis models, it downregulates the Th17-IL-17 axis and shifts macrophage polarization (31, 32). Nintedanib similarly rebalances macrophage phenotype away from the profibrotic M2 state (33, 34). The message is clear—different FGF family members can either amplify or dampen immune activation, and the net immunological effect depends entirely on which ligand-receptor pair dominates in a given tissue context.

### 3.3 Angiogenesis, hypoxia, and endothelial activation

Angiogenesis is a hallmark of chronic inflammation in rheumatic diseases, sustaining nutrient supply to hyperplastic synovium, fibrotic tissue, and entheseal inflammatory infiltrates (28). FGF2 is one of the most potent pro-angiogenic factors, acting synergistically with VEGF to promote endothelial cell proliferation, migration, and tube formation (35). In the RA synovium, FGF2 cooperates with hypoxia-inducible pathways to sustain neovascularization (36). In SpA, FGF2-mediated angiogenesis has been linked to angiogenesis–osteogenesis coupling, a process whereby new blood vessel formation facilitates subsequent endochondral bone formation at entheseal sites (37). FGFR1 SUMOylation has been identified as a regulatory mechanism coordinating endothelial angiogenic signaling, suggesting additional layers of post-translational control (38). The dual role of FGF2 in promoting both angiogenesis and fibroblast activation creates a feed-forward loop that may sustain chronic tissue remodeling in multiple rheumatic diseases.

### 3.4 Extracellular matrix remodeling, matricellular proteins, and EMT-like changes

FGF/FGFR signaling modulates extracellular matrix (ECM) dynamics through both proteolytic and non-proteolytic mechanisms. In RA-FLS, FGF2 stimulation increases MMP-1, MMP-3, and MMP-13 expression, thereby promoting cartilage and bone matrix degradation (16, 39). In fibrotic contexts, FGF signals can promote epithelial-to-mesenchymal transition (EMT)-like changes, contributing to the expansion of myofibroblast populations in SSc skin and lung (40). Pentraxin 3 (PTX3) has been identified as a natural antagonist of FGF2-mediated ECM remodeling, inhibiting FGF2-induced osteoclastogenesis and matrix degradation in RA (41).

Beyond MMPs and PTX3, FGF/FGFR signaling should be interpreted within the broader ECM niche. Matricellular proteins such as periostin, CCN2/CTGF, tenascin-C, and osteopontin are not passive structural components of the ECM; rather, they regulate growth factor availability, integrin signaling, mechanotransduction, fibroblast activation, inflammatory cell retention, and tissue repair responses (42–49). These molecules may influence whether FGF signaling promotes regenerative repair, persistent fibroblast activation, angiogenesis, or fibrosis. For example, CCN2 and periostin are closely linked to fibrotic matrix remodeling and TGF-β-associated fibroblast activation, whereas tenascin-C and osteopontin can influence inflammatory matrix organization and leukocyte–stromal interactions. Although direct FGF/FGFR-specific evidence in autoimmune rheumatic tissues remains limited, future studies should examine FGF ligand–receptor pairs together with ECM-derived modifiers rather than treating FGF signaling as an isolated pathway.

This expanded ECM perspective is particularly relevant to SSc fibrosis, RA pannus invasion, SpA/PsA entheseal remodeling, and Sjögren’s glandular repair, where the matrix environment may determine whether FGF signaling drives damage or regeneration.

### 3.5 Bone remodeling: erosion and new bone formation

FGF/FGFR signaling regulates both sides of the bone remodeling equation. On the resorptive side, FGF2 promotes osteoclast maturation through upregulation of RANKL expression on synovial fibroblasts and induction of osteoclast precursor differentiation (16, 41). FGF2 binding to heparan sulfate proteoglycans on RA synovial fibroblasts induces RANKL expression and enhances osteoclast maturation, contributing directly to periarticular bone erosion (50). On the formative side, FGF signaling participates in endochondral ossification, the process responsible for pathological new bone formation at entheseal sites in SpA and PsA (37, 51). FGF7/FGFR2IIIb signaling has recently been identified as a mediator of ankylosing enthesitis in PsA, potentially bridging IL-17-driven inflammation and structural damage (52). FGF18/FGFR3 signaling promotes chondrocyte differentiation and has been explored therapeutically through intra-articular sprifermin for cartilage repair (53).

### 3.6 Cross-disease comparison: shared mechanisms, divergent outcomes

A key insight from examining FGF/FGFR signaling across rheumatic diseases is that the same upstream signals can produce vastly different tissue outcomes depending on the cellular context and disease microenvironment. FGF2/FGFR3 signaling in the RA synovium drives FLS proliferation and bone erosion, whereas in SSc it contributes to fibroblast activation and extracellular matrix deposition, and in SpA it mediates angiogenesis–osteogenesis coupling and new bone formation. These divergent outcomes reflect differences in the downstream effector cells (FLS versus myofibroblasts versus entheseal progenitors), the local cytokine milieu (TNF/IL-6-dominant versus TGF-β-dominant versus IL-17-dominant), and the tissue architecture (synovial versus pulmonary versus entheseal) (2, 3).

The endocrine FGF23/Klotho axis presents a different pattern: it appears elevated across multiple diseases (RA, SLE, SSc, SpA) but may reflect shared pathophysiological processes—systemic inflammation, metabolic disturbance, and cardiovascular risk—rather than disease-specific mechanisms (11–13, 54). This “convergent biomarker” pattern contrasts with the “divergent effector” pattern of paracrine FGFs, and has important implications for therapeutic targeting: paracrine FGF axes may require disease-specific and tissue-specific intervention, while endocrine FGF axes may respond to more generalizable approaches.

**Figure 2:**
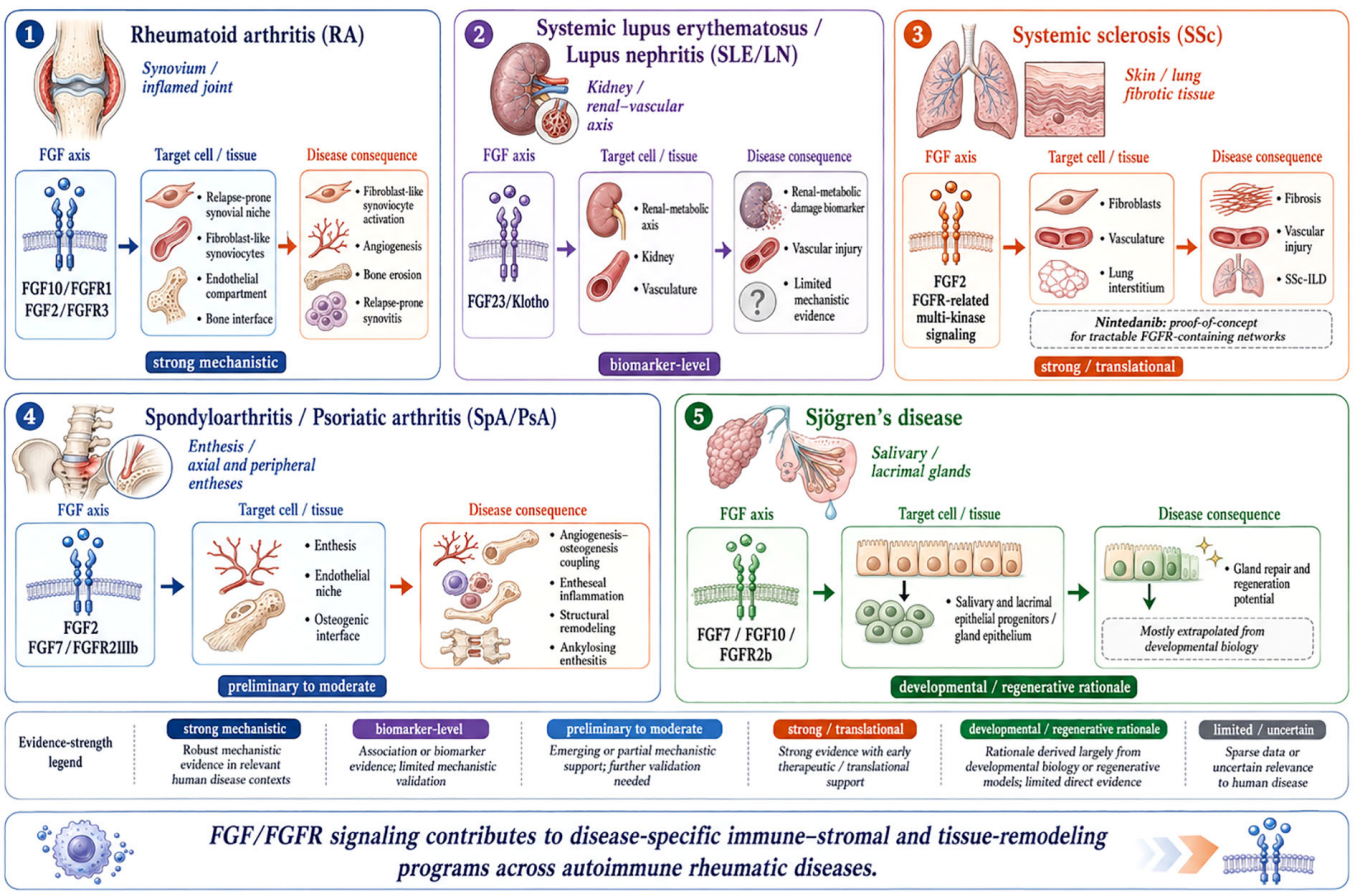
Disease-specific FGF/FGFR axes and pathological implications across autoimmune rheumatic diseases. FGF/FGFR signaling contributes to autoimmune rheumatic diseases through distinct disease- and tissue-specific axes. In rheumatoid arthritis, FGF10/FGFR1 and FGF2/FGFR3 signaling are implicated in relapse-prone synovial niches, fibroblast-like synoviocyte activation, angiogenesis, and bone erosion. In systemic lupus erythematosus and lupus nephritis, FGF23/Klotho signaling is best interpreted as an emerging biomarker axis reflecting renal-metabolic injury, vascular risk, and damage accrual rather than a validated inflammatory driver. In systemic sclerosis, FGF-related signaling participates in fibroblast activation, vascular injury, and fibrotic remodeling, while nintedanib provides clinical evidence that multi-kinase pathways including FGFR-related signaling are therapeutically tractable in SSc-associated interstitial lung disease. In spondyloarthritis and psoriatic arthritis, FGF2-mediated angiogenesis-osteogenesis coupling and FGF7/FGFR2IIIb signaling may contribute to entheseal inflammation and aberrant new bone formation. In Sjögren’s disease, FGF7/FGF10/FGFR2b signaling currently has greatest translational relevance for salivary and lacrimal gland repair, based primarily on developmental and regenerative biology evidence. Visual evidence coding is used to distinguish human mechanistic evidence, preclinical or biomarker-level evidence, extrapolated evidence, and clinical translational evidence. The image emphasizes that evidence strength varies substantially across diseases.

What ties these disparate mechanisms together? The convergence of fibroblast activation, immune modulation, angiogenesis, matrix remodeling, and bone metabolism positions FGF/FGFR signaling as a multifunctional node at the immune-stromal interface. It is not a single-purpose inflammatory mediator. Rather, the FGF system integrates diverse tissue responses to chronic inflammation—which is precisely why its pathological roles vary so markedly across diseases. The disease-specific sections that follow examine how these general mechanisms play out in each clinical context (**Figure 2**; **Table 2**).

**Table 2:** Disease-specific evidence appraisal of FGF/FGFR signaling.

Disease	FGF/FGFR axis	Main tissue/process	Evidence level	Key limitation	Translational implication
RA	FGF10/FGFR1	Relapse-prone synovium; CD55^+^ lining FLS	Strong mechanistic evidence	Limited cohorts; needs multicenter replication and relapse prediction	Synovial relapse-risk biomarker; local modulation as proof-of-mechanism concept
RA	FGF2/FGFR3	FLS activation, angiogenesis, osteoclastogenesis	Strong mechanistic evidence	Extensive experimental data but no clinical FGFR-targeted validation	Target validation for synovial pharmacodynamic studies
RA	FGF23/Klotho	Renal-metabolic and bone/cardiovascular comorbidity markers	Biomarker-level evidence	Cross-sectional serum studies; no synovial tissue causality	Potential adjunct biomarker only after longitudinal validation
RA	FGF21	CIA immune modulation and cartilage protection	Moderate mechanistic evidence	Preclinical arthritis models only; no human autoimmune trial data	Hypothesis for metabolic-immune studies, not a validated RA therapy
SLE/LN	FGF23/Klotho	Renal-metabolic damage and vascular-risk markers	Biomarker-level evidence	Unclear whether mediator or secondary marker of renal impairment	Longitudinal renal-risk biomarker candidate
SLE/LN	FGF21/Klotho-related pathways	Murine lupus nephritis; inflammasome and renal injury	Preliminary/extrapolated evidence	Single murine or indirect genetic studies; limited tissue validation	Mechanistic hypothesis requiring renal tissue confirmation
SSc	FGF2	Skin/lung fibroblasts; fibrosis and repair	Moderate mechanistic evidence	Dual pro- and anti-fibrotic effects depend on cell type and stage	Stage- and cell-type-specific pathway mapping before intervention
SSc	FGF23/Klotho	Vascular-metabolic burden	Biomarker-level evidence	Small cross-sectional cohorts and conference-level evidence	Composite vascular-metabolic biomarker candidate
SSc-ILD	FGFR-containing multi-kinase network (nintedanib)	Progressive fibrosing ILD	Clinical translational evidence	Nintedanib is not FGFR-selective; FGFR-specific causality unresolved	Clinically tractable multi-kinase pathway; target attribution studies needed
SpA/PsA	FGF2/ATF6	Entheseal angiogenesis-osteogenesis coupling	Moderate mechanistic evidence	Animal/*in vitro* support with limited direct human enthesis validation	Candidate structural-progression mechanism
PsA	FGF7/FGFR2IIIb	Ankylosing enthesitis in PsA-related models	Preliminary/extrapolated evidence	Single study; human entheseal tissue validation missing	Candidate mediator for focused replication studies
SpA/AS	FGF23/Klotho	Peripheral biomarker; inflammation, bone, vascular risk	Biomarker-level evidence	Low disease specificity; no prediction of structural progression	Peripheral marker requiring longitudinal imaging correlation
Sjögren’s disease	FGF7/FGF10/FGFR2b	Salivary/lacrimal epithelial differentiation and gland repair	Preliminary/extrapolated evidence	Developmental/organoid data; no autoimmune gland efficacy	Regenerative rationale requiring patient-tissue and disease-model validation

## 4 Rheumatoid arthritis: synovial remodeling, relapse and bone erosion

Rheumatoid arthritis provides the most mechanistically developed evidence for FGF/FGFR involvement among autoimmune rheumatic diseases. Three distinct FGF axes have been implicated in RA pathogenesis, each operating at a different level—local synovial signaling (FGF10/FGFR1 and FGF2/FGFR3) versus systemic endocrine signaling (FGF23/Klotho)—with markedly different evidence strength and translational implications.

### 4.1 FGF10/FGFR1 and relapse-prone synovial niches

A landmark study by Meng et al. (2024) identified FGF10/FGFR1 signaling as a critical driver of relapse-prone synovial niches in RA (9). Using single-cell RNA sequencing of synovial tissue from RA patients in clinical remission who subsequently relapsed versus those who maintained remission, the authors demonstrated that CD55^+^ lining fibroblast-like synoviocytes (FLS) expressing high levels of FGF10 persisted in the synovium of relapse-prone patients. FGF10 signaling through FGFR1 maintained these cells in an activated, proliferation-competent state, effectively creating a “primed” synovial niche that could rapidly reactivate upon treatment withdrawal (9, 55).

This finding carries several important implications. First, it demonstrates that FGF signaling can operate independently of active systemic inflammation, functioning as a tissue-autonomous persistence mechanism. Second, it provides a potential biomarker for relapse risk stratification: synovial FGF10/FGFR1 expression at the time of remission could theoretically identify patients who are at high risk of flare upon treatment de-escalation. Third, it raises a testable therapeutic hypothesis that local FGFR1 modulation could be explored as an adjunct strategy in relapse-prone RA, provided that safety and synovial pharmacodynamic effects are demonstrated. The Nature Reviews Rheumatology commentary by McHugh (2023) highlighted this work as evidence that FGF signaling is directly implicated in the biology of relapsing RA (55).

FGFR1 has also been identified as a potential marker of terminal effector peripheral helper T cells in RA, suggesting that FGFR1 expression on both stromal and immune cells may contribute to the persistence of synovial inflammation (56).

### 4.2 FGF2/FGFR3 axis in FLS proliferation and joint destruction

The FGF2 (bFGF)/FGFR3 axis is, by volume of evidence, the best-characterized FGF pathway in RA. Two decades of work have established that FGF2 is abundantly expressed in the RA synovium and synovial fluid, where it promotes FLS proliferation, migration, and tissue invasion through FGFR3-mediated MAPK/ERK signaling (16, 57). But FGF2’s role extends well beyond FLS biology.

In the rheumatoid joint, FGF2 participates in a pathogenic triad involving synovial angiogenesis, FLS activation, and osteoclastogenesis. It drives pannus neovascularization alongside VEGF, sustaining the oxygen and nutrient demands of the hyperplastic synovium (36, 58). It activates FLS through the FGFR3-RSK2 signaling axis—a pathway that has proven pharmacologically tractable in experimental settings, as kaempferol (a natural flavonoid) and proteasome inhibitors suppress FLS pathology by targeting this cascade (23, 57). It also promotes osteoclastogenesis by inducing RANKL expression on synovial fibroblasts via heparan sulfate proteoglycan-mediated binding, directly contributing to periarticular bone erosion (50). The breadth of this involvement is captured in the recent integrative review by Illanad et al. (2026), which discusses FGF2 as a prominent contributor within the pathogenic microenvironment of RA (16).

What restrains FGF2 in healthy tissue? Pentraxin 3 (PTX3) appears to be a key endogenous brake. PTX3 directly binds FGF2, sequestering it from FGFR engagement and thereby inhibiting FGF2-induced osteoclast formation (41). This FGF2/PTX3 balance within the synovial microenvironment may determine erosion severity—a concept that merits clinical validation as a local tissue biomarker. Novel extracellular FGF2/FGFR inhibitors identified through computational screening further expand the toolbox for pathway modulation (59).

More recent work has begun to connect FGF2 signaling to emerging themes in RA biology. The E3 ubiquitin ligase CUL4A promotes FLS glycolytic metabolism by targeting FGF2 for proteasomal degradation, linking this axis to immunometabolism (60). MicroRNA-mediated regulation (miR-653-5p) and post-translational control via Roquin-1/Regnase-1 add additional layers of complexity (61, 62). Meanwhile, novel 3D spheroid co-culture models incorporating FLS, macrophages, and endothelial cells now enable the study of FGF-mediated immune-stromal interactions in a tissue-relevant context (63). Collectively, these studies position FGF2/FGFR3 not as a single target but as a multifaceted signaling node operating at the intersection of FLS biology, angiogenesis, bone metabolism, and immunometabolism.

### 4.3 FGF23/Klotho in RA: biomarker or bystander?

The evidence for FGF23/Klotho in RA tells a very different story. Here, the data are primarily correlative. Elevated serum FGF23 has been reported in RA patients, correlating with inflammatory markers, bone mineral density, lipid profiles, and cardiovascular risk (11, 12). One study offers a mechanistic bridge: Hu et al. (2024) showed that FGF23 facilitates IL-1β synthesis in RA through PI3K/AKT/NF-κB activation (24), and Ji et al. (2025) demonstrated that Klotho promotes bone formation in RA-associated osteoporosis (64). Yet these findings must be interpreted cautiously.

The fundamental question is whether FGF23 elevation in RA reflects joint-specific pathobiology or is simply a systemic marker of the metabolic and cardiovascular comorbidity burden. Most studies are cross-sectional, precluding causal inference. Synovial tissue-level data for FGF23/Klotho are essentially absent. Until prospective studies demonstrate that FGF23 predicts RA outcomes independently of conventional markers, it remains an intriguing but unvalidated biomarker candidate.

FGF21 represents an intriguing counterpoint within the endocrine FGF subfamily. Multiple studies in CIA models have demonstrated anti-inflammatory and joint-protective effects of FGF21, including downregulation of the Th17-IL-17 axis, enhancement of regulatory T cell function, and amelioration of cartilage damage (31, 32, 65). In preclinical CIA models, FGF21 showed anti-arthritic effects in experimental settings that included comparison with established anti-inflammatory therapy, but these findings remain far from clinical validation (66). FGF21 also enhanced the therapeutic efficacy of dexamethasone while reducing its side effects in experimental arthritis (67). Whether these preclinical findings translate to human RA remains to be determined.

In RA, local synovial FGF/FGFR signaling—particularly the FGF10/FGFR1 and FGF2/FGFR3 axes—appears more mechanistically relevant and therapeutically actionable than circulating endocrine FGF biomarkers. Future studies should prioritize tissue-level validation of FGF23/Klotho and explore whether FGF21 agonism offers therapeutic potential in RA.

Evidence appraisal and translational readiness. Among the diseases reviewed here, RA possesses the strongest mechanistic evidence for FGF/FGFR involvement. The FGF10/FGFR1 axis is supported by human synovial tissue-level scRNA-seq data with functional validation (strong mechanistic evidence). The FGF2/FGFR3 axis is supported by extensive *in vitro* FLS studies, animal models, and pharmacological target validation through kaempferol and proteasome inhibitor studies (strong mechanistic evidence). The FGF23/Klotho axis in RA is limited to cross-sectional serum biomarker studies without tissue-level validation (biomarker-level evidence). FGF21 therapeutic effects are demonstrated in CIA animal models but lack human autoimmune data (moderate mechanistic evidence). In the context of this review, RA serves as the benchmark disease in which FGF/FGFR axes have moved beyond peripheral biomarker association toward disease-tissue mechanisms. However, even in RA, therapeutic translation should remain cautious. The immediate next step is not chronic systemic FGFR inhibition, but validation of synovial target engagement, cell-type specificity, and relapse-associated pharmacodynamic readouts in defined patient subgroups.

## 5 Systemic lupus erythematosus and lupus nephritis: FGF23/Klotho as a renal-metabolic biomarker axis

Unlike RA, where paracrine FGF axes have tissue-level mechanistic support, evidence for FGF/FGFR involvement in SLE/LN is concentrated mainly on the endocrine FGF23/Klotho axis. This axis should currently be interpreted as an emerging renal-metabolic and inflammatory biomarker axis rather than a validated therapeutic target. Importantly, the reported association between FGF23 and urinary MCP-1/CCL2 in active LN suggests a plausible link between mineral-metabolic stress and renal chemokine-mediated inflammation, but this relationship requires renal tissue-level and longitudinal validation before causal conclusions can be drawn.

### 5.1 FGF23 as a renal-metabolic and inflammatory biomarker

The strongest SLE/LN evidence concerns circulating FGF23. Serum FGF23 levels are increased in patients with active LN and have been associated with renal involvement, proteinuria, reduced kidney function, inflammatory markers, and urinary MCP-1/CCL2 (68–73). Because urinary MCP-1/CCL2 reflects renal chemokine activity and inflammatory cell recruitment, the FGF23-MCP-1 association raises a testable hypothesis: FGF23 elevation in LN may capture not only impaired mineral metabolism or kidney function but also a renal inflammatory microenvironment (71–73). However, current evidence remains insufficient to determine whether FGF23 actively contributes to LN pathogenesis, marks renal tubular or vascular stress, or simply reflects renal impairment and systemic inflammation. Therefore, FGF23 should be positioned as a candidate prognostic or stratification biomarker, not as a validated inflammatory driver.

### 5.2 α-Klotho deficiency and its implications

The other side of this axis—α-Klotho—has attracted attention as a potential independent biomarker. Reduced circulating Klotho levels in SLE correlate with higher disease activity and organ damage (74), and soluble α-Klotho has even been proposed as a biomarker for neuropsychiatric SLE (75). The therapeutic angle is more speculative but intriguing: Klotho supplementation in murine lupus nephritis reduced blood pressure and albuminuria (76), hinting at immunomodulatory roles that extend beyond the classical metabolic framework. Whether this murine observation has human relevance remains entirely open.

### 5.3 FGF21 and non-canonical FGF pathways in SLE

Beyond the FGF23/Klotho axis, the evidence thins considerably. A single murine study demonstrated that FGF21 mitigates lupus nephritis progression through the Irgm1/NLRP3 inflammasome pathway (77). MicroRNA-199a has been implicated in lupus nephritis pathogenesis through modulation of Klotho expression (78), and a Mendelian randomization study has provided suggestive—though far from conclusive—evidence for a causal link between genetically predicted Klotho levels and autoimmune disease risk (79, 80). These scattered findings do not yet constitute a coherent mechanistic narrative.

### 5.4 Limitations and interpretation

Several important limitations constrain the interpretation of FGF23/Klotho findings in SLE. Most studies are cross-sectional with small sample sizes, precluding determination of whether FGF23 elevation is a cause, consequence, or epiphenomenon of renal damage. FGF23 is a well-established biomarker of chronic kidney disease (CKD) progression regardless of etiology, raising the question of whether its elevation in lupus nephritis reflects SLE-specific pathobiology or simply the degree of renal impairment. The absence of renal tissue-level data for FGF23/Klotho expression in lupus nephritis limits mechanistic interpretation.

In SLE, FGF23/Klotho signaling should currently be interpreted primarily as a biomarker axis reflecting renal, metabolic, and damage-related processes rather than a validated inflammatory driver. Prospective longitudinal studies with serial FGF23/Klotho measurements and renal outcomes are needed to determine whether this axis has prognostic value independent of conventional markers of renal function.

Future validation in SLE/LN should proceed in three steps. First, longitudinal cohorts should measure serum FGF23, soluble Klotho, urinary MCP-1/CCL2, proteinuria, eGFR, complement, anti-dsDNA, and treatment response at prespecified time points to determine whether the FGF23/Klotho axis adds prognostic value beyond conventional renal markers. Second, renal biopsy-based studies should map FGFRs, Klotho expression, MCP-1/CCL2, tubular injury markers, endothelial activation, and macrophage infiltration using spatial transcriptomics, multiplex immunostaining, or single-nucleus approaches. Third, mechanistic models should test whether FGF23 directly modulates renal tubular, endothelial, mesangial, or immune-cell chemokine programs under inflammatory conditions. Only if these steps demonstrate tissue localization, temporal association, and functional causality should therapeutic modulation of this axis be considered.

Evidence appraisal and translational readiness. The SLE/LN evidence remains primarily biomarker-level, based on small clinical cohorts and limited mechanistic validation. The association of FGF23 with urinary MCP-1/CCL2 provides an important inflammatory clue, but it does not establish renal tissue causality. At present, the FGF23/Klotho axis is most appropriate for longitudinal biomarker validation and renal tissue mapping. It is not yet appropriate for therapeutic targeting in human SLE/LN. The FGF21/Irgm1/NLRP3 and Klotho supplementation data remain preliminary because they derive mainly from murine models and require confirmation in human tissue or patient-derived systems.

## 6 Systemic sclerosis: fibrosis, vascular injury and antifibrotic translation

Systemic sclerosis presents the most clinically advanced translational context for FGFR-containing multi-kinase pathway modulation, largely owing to the efficacy of nintedanib in SSc-associated interstitial lung disease (SSc-ILD). The evidence in SSc spans three domains: FGF signaling in fibroblast activation and fibrosis, FGF23/Klotho as a vascular-metabolic biomarker, and the translational lessons from nintedanib.

### 6.1 FGF signaling in fibroblast activation and tissue fibrosis

The relationship between FGF2 and fibrosis in SSc defies simple categorization. This is not a straightforward pro-fibrotic signal. FGF2 has been implicated in both driving and opposing fibrosis, and the direction appears to depend on the tissue context, disease stage, and cellular target (5, 30).

On the pro-fibrotic side, FGF2 can promote fibroblast proliferation and extracellular matrix deposition through FGFR-mediated MAPK/ERK activation in SSc skin fibroblasts. However, the matrix profile it induces differs qualitatively from the classical TGF-β-driven fibrotic response (81)—a distinction that may prove therapeutically important. On the anti-fibrotic side, FGF2 has been shown to counteract TGF-β-induced myofibroblast differentiation (30), and the stromal vascular fraction mitigates bleomycin-induced skin fibrosis in part by modulating FGF-related signaling (82). An FGF2-derived short peptide attenuated bleomycin-induced pulmonary fibrosis by blocking the fibroblast-to-myofibroblast transition (83). Single-cell analyses of SSc tissue have revealed disease-specific alterations in FGF-mediated paracrine communication between adipose-derived stem cells, fibroblasts, and endothelial cells (84), further underscoring the complexity.

The therapeutic implication of this duality is sobering. Block FGF2 indiscriminately, and protective reparative signals may be lost. Augment it, and fibroblast activation could be amplified. This paradox makes SSc a disease context in which cell-type-specific and stage-specific understanding of FGF signaling is particularly important.

### 6.2 FGF23/Klotho and vascular-metabolic injury in SSc

The FGF23/Klotho axis in SSc follows the now-familiar biomarker pattern seen across rheumatic diseases—but with a vascular twist. The FGF23-to-α-Klotho index correlates with SSc disease activity, skin thickness, and anti-topoisomerase I antibody positivity (13). Given the centrality of vasculopathy to SSc pathogenesis, FGF23/Klotho may be capturing a dimension of vascular-metabolic injury that conventional markers miss—though, as in other diseases, the evidence remains small-scale and cross-sectional (85).

The interpretation of FGF23/Klotho in SSc faces similar challenges as in other rheumatic diseases: small sample sizes, cross-sectional designs, and the difficulty of distinguishing disease-specific effects from those related to comorbidities such as renal dysfunction and cardiovascular disease. Nevertheless, FGF23/Klotho may serve as a composite marker of the vascular-metabolic burden that characterizes SSc.

### 6.3 Nintedanib and the translational lessons from SSc-ILD

The approval of nintedanib for SSc-ILD represents the most significant translational milestone for FGF-related signaling in autoimmune rheumatic diseases. The SENSCIS trial (Distler et al., 2019) demonstrated that nintedanib reduced the annual rate of forced vital capacity (FVC) decline in patients with SSc-ILD compared to placebo, leading to regulatory approval for this indication (10). This trial, along with the earlier INPULSIS trials in idiopathic pulmonary fibrosis (IPF), established that multi-kinase pathways including FGFR-related signaling can be clinically targeted in fibrosing lung disease (86).

However, a critical caveat shapes the interpretation of this success. Nintedanib is not a selective FGFR inhibitor. It is a triple angiokinase inhibitor targeting FGFR1–3, VEGFR1–3, and PDGFR-α/β (87). This means its clinical benefit cannot be attributed to FGFR blockade alone. Preclinical work has attempted to tease apart the contributions: nintedanib inhibits both fibroblast activation and macrophage polarization in SSc models, with effects that correlate with FGFR-related pathway modulation (33, 34, 88, 89). But correlation is not causation. Without selective FGFR inhibitor comparators, the question of “how much of nintedanib’s effect comes from FGFR?” remains unanswered.

The real-world experience with nintedanib has been largely reassuring. Multiple cohorts confirm its tolerability in SSc-ILD, though gastrointestinal side effects—particularly diarrhea—remain the primary limitation (90–92). Benefits have been observed across connective tissue disease subtypes with progressive fibrosing ILD, including RA-ILD (93–97).

The nintedanib experience provides three key translational lessons. First, FGFR-containing multi-kinase networks are clinically tractable in SSc-ILD, but FGFR-specific causality remains unresolved. Second, the safety profile of chronic FGFR-containing pathway modulation in a non-oncology population is manageable, though gastrointestinal toxicity remains a significant limitation. Third, the inability to dissect FGFR-specific contributions from nintedanib’s multi-target effects creates a need for studies using selective FGFR inhibitor comparators, target-engagement biomarkers, or biomarker-stratified analyses to determine whether any patient subgroup derives benefit specifically from FGFR blockade (**Table 3**).

**Table 3:** Therapeutic agents related to FGF/FGFR pathways and clinical status.

Agent	Target	Approved indication/trial context	Clinical status in autoimmune rheumatic disease	Relevance to rheumatic disease	Opportunities	Safety concerns
Nintedanib	FGFR1-3, VEGFR, PDGFR	IPF; SSc-ILD	Clinically approved/validated in SSc-ILD	Directly relevant to SSc-ILD but not FGFR-selective	Antifibrotic multi-kinase pathway; responder biomarker studies	GI side effects; liver monitoring; FGFR-specific causality unresolved
Erdafitinib	Pan-FGFR1-4	Urothelial carcinoma	Oncology-approved; no autoimmune clinical validation	Safety data informative only	Research tool for target-engagement studies	Hyperphosphatemia; ocular toxicity; mucositis
Pemigatinib	FGFR1-3	Cholangiocarcinoma	Oncology-approved; no autoimmune clinical validation	Selective FGFR inhibition concept	Preclinical pathway-dissection tool	Hyperphosphatemia; nail toxicity; ocular risk
Futibatinib	Irreversible pan-FGFR1-4	Cholangiocarcinoma	Oncology-approved; no autoimmune clinical validation	Irreversible inhibition model	Mechanistic comparator only	Pan-FGFR class toxicities
Burosumab	Anti-FGF23 monoclonal antibody	X-linked hypophosphatemia	Approved for XLH; no autoimmune clinical validation	Shows ligand-level FGF23 neutralization is feasible	Only for a clearly pathogenic FGF23 subgroup, if identified	Ectopic mineralization and phosphate-monitoring concerns
FGF21 analogues	FGF21/FGFR1c/β-Klotho	MASH/NASH metabolic disease trials	Metabolic disease trials; no autoimmune clinical validation	Preclinical CIA anti-inflammatory signal only	Metabolic-immune comorbidity studies	GI effects, weight loss; autoimmune efficacy unproven
Sprifermin (rhFGF18)	FGFR3 agonist	Knee osteoarthritis trials	OA trials; no autoimmune inflammatory arthritis validation	Proof-of-concept for local FGF delivery to joint tissues	Cartilage-repair biology; local delivery model	Joint-specific delivery; not an RA disease-modifying therapy
Experimental anti-FGFR2IIIb strategies	FGFR2IIIb	Preclinical/early clinical oncology context	No autoimmune clinical validation	Candidate PsA enthesis pathway only	Isoform-selective mechanistic testing	Limited safety data; epithelial repair concerns

Evidence appraisal and translational readiness. SSc provides clinical translational evidence through nintedanib’s efficacy in SSc-ILD. However, nintedanib is not FGFR-selective, so the FGFR-specific contribution remains unresolved. The FGF2 dual role in fibrosis is supported by *in vitro* and animal model data but complicated by context-dependency (moderate mechanistic evidence). FGF23/Klotho as a vascular-metabolic biomarker in SSc remains preliminary/extrapolated evidence with limited sample sizes. The key translational gap is the absence of selective FGFR inhibitor data in SSc models or patients.

## 7 Spondyloarthritis and psoriatic arthritis: preliminary links to enthesitis and aberrant bone formation

The SpA spectrum—including axial spondyloarthritis (axSpA), ankylosing spondylitis (AS), and psoriatic arthritis (PsA)—is distinguished from other rheumatic diseases by the paradoxical coexistence of bone erosion and pathological new bone formation, particularly at entheseal sites (98). FGF signaling is emerging as a potential mediator of this transition from inflammation to structural remodeling, though the evidence base remains less developed than in RA or SSc.

### 7.1 FGF23 in axSpA: a limited peripheral biomarker

As in RA and SLE, serum FGF23 is elevated in axSpA, correlating with inflammatory markers and radiographic spinal damage (54). But the pattern by now is familiar—and the limitations equally so. Without entheseal tissue-level data, circulating FGF23 in SpA almost certainly reflects systemic inflammation and metabolic disturbance rather than anything specific to the entheseal disease process. Klotho levels in SpA patients have been linked to subclinical atherosclerosis (99, 100), reinforcing the cardiovascular–metabolic dimension, but no longitudinal study has tested whether FGF23 predicts structural progression.

### 7.2 FGF2 and angiogenesis–osteogenesis coupling

The mechanistically more interesting story in SpA involves FGF2 at the enthesis. In an elegant series of studies, Ma et al. demonstrated that endoplasmic reticulum stress in entheseal cells drives FGF2 secretion through the ATF6 transcription factor, promoting both new blood vessel formation and subsequent endochondral bone formation (37). This angiogenesis–osteogenesis coupling provides a plausible mechanism for the signature pathological feature of SpA: new bone formation at inflammatory sites. The same group identified a reinforcing loop: SMURF2-mediated degradation of PTX3 releases FGF2 from its endogenous brake, amplifying angiogenesis (101).

Does this process operate in human disease? Indirect evidence suggests so. PsA synovial fibroblasts exhibit enhanced angiogenic function with increased FGF2 expression compared to osteoarthritis controls (102), and altered levels of FGF family members have been detected in the serum of psoriasis patients—potentially reflecting early FGF-mediated angiogenic dysregulation before overt musculoskeletal involvement (103).

### 7.3 FGF7/FGFR2IIIb and entheseal ossification in PsA

Perhaps the most novel contribution to understanding FGF signaling in SpA comes from Ebihara et al. (2025), who proposed FGF7/FGFR2IIIb as a candidate mediator of ankylosing enthesitis in PsA-related models (52). This study demonstrated that FGF7 signaling through the FGFR2IIIb splice variant in entheseal stromal cells can drive pathological bone formation through mechanisms that connect IL-17-driven inflammation with structural remodeling. This finding is significant because it implicates a specific FGF ligand-receptor pair (FGF7/FGFR2IIIb) in a disease process that has traditionally been attributed primarily to BMP and Wnt signaling (104).

The relationship between FGF and other bone-forming pathways warrants further investigation. FGF signaling intersects with BMP and Wnt pathways at multiple levels during endochondral ossification, and the relative contributions of these pathways to entheseal new bone formation in SpA remain to be fully elucidated (51). Li et al. (2025) recently reported distinct serum cytokine and chemokine profiles in AS versus non-radiographic axSpA, including differences in FGF-related markers, suggesting heterogeneity in the role of FGF signaling across the SpA spectrum (105).

In SpA and PsA, FGF signaling may be more relevant to the transition from inflammation to structural remodeling than to systemic inflammation itself. The identification of FGF7/FGFR2IIIb and FGF2/ER stress/angiogenesis axes provides testable mechanistic hypotheses, but direct validation in human entheseal tissue remains the critical missing evidence before these axes can be considered disease-relevant in human SpA/PsA.

Evidence appraisal and translational readiness. In SpA/PsA, FGF2-mediated angiogenesis-osteogenesis coupling and FGF7/FGFR2IIIb signaling should be considered candidate mechanisms for the transition from entheseal inflammation to structural remodeling, not established mechanisms in human disease. Direct validation in human entheseal tissue, imaging-linked longitudinal cohorts, and functional testing in disease-relevant stromal or osteochondral models are required before therapeutic implications can be inferred.

## 8 Sjögren’s disease: regenerative rationale from glandular biology rather than established disease-specific evidence

Among the diseases reviewed here, Sjögren’s disease has the weakest direct evidence linking FGF/FGFR signaling to autoimmune pathogenesis. However, the well-established role of FGF signaling in salivary gland development and regeneration provides a distinct translational angle: FGF-supported glandular repair rather than immunosuppression.

### 8.1 FGF/FGFR signaling in salivary gland biology

FGF7, FGF10, and their shared receptor FGFR2IIIb are master regulators of salivary gland branching morphogenesis during development (106). Crucially, this is not merely a developmental relic. FGFR2 remains essential for duct homeostasis and acinar cell differentiation in adult mice (107), and a mesenchymal-to-epithelial switch in FGF10 expression specifies an evolutionarily conserved progenitor population in the adult gland (108). The machinery for FGF-driven glandular repair, in other words, persists into adulthood—at least in healthy tissue (109).

### 8.2 Regenerative medicine applications

This persistent regenerative capacity has been exploited in increasingly sophisticated organoid models. FGF signaling has emerged as a key mediator of stromal-epithelial communication in salivary gland organoids, with PDGFRα^+^ stromal subpopulations promoting proacinar differentiation through FGF-dependent mechanisms (110). Retinoic acid and FGF10 together can drive pluripotent stem cells toward salivary gland-like organoids (111), and FGF2 serves as an essential niche factor in mesenchyme-dependent organoid systems (112). More advanced bioengineering approaches—including innervated secretory organoids created through magnetic 3D bioprinting (113) and combination strategies using laminin peptides with FGF9 (114)—suggest experimental feasibility of FGF-supported glandular epithelial differentiation, but they do not yet establish therapeutic efficacy in autoimmune-mediated glandular destruction.

### 8.3 Bridging developmental biology to Sjögren’s therapeutics

The translation of these developmental and regenerative findings to Sjögren’s disease therapeutics faces several challenges. The destructive inflammatory process in Sjögren’s disease may damage or deplete the FGF-responsive progenitor cells needed for regeneration. No studies have directly evaluated FGF-based regenerative therapies in Sjögren’s disease models. The relationship between salivary FGF levels (such as FGF4 identified in proteomic studies) and disease activity or glandular function remains poorly characterized.

In Sjögren’s disease, FGF signaling may have greater translational relevance for epithelial repair and glandular regeneration than for systemic immune suppression. Future studies should first determine whether FGF-responsive progenitor or epithelial cell populations persist in autoimmune-damaged glands, whether FGF-supported differentiation can restore function under inflammatory conditions, and whether salivary FGF profiles can serve as biomarkers of residual regenerative capacity.

Evidence appraisal and translational readiness. In Sjögren’s disease, the FGF7/FGF10/FGFR2b axis provides a biologically plausible regenerative hypothesis, but the evidence is extrapolated mainly from developmental biology, adult gland homeostasis, and organoid systems. These data do not establish that FGF-responsive progenitor or epithelial populations persist in autoimmune-damaged glands, nor that FGF supplementation can restore function under chronic inflammatory conditions. Patient-derived organoids, inflamed gland models, and biopsy-based mapping of residual FGF-responsive cell populations should therefore precede therapeutic speculation.

## 9 Translational prioritization of FGF/FGFR axes

**Figure 3:**
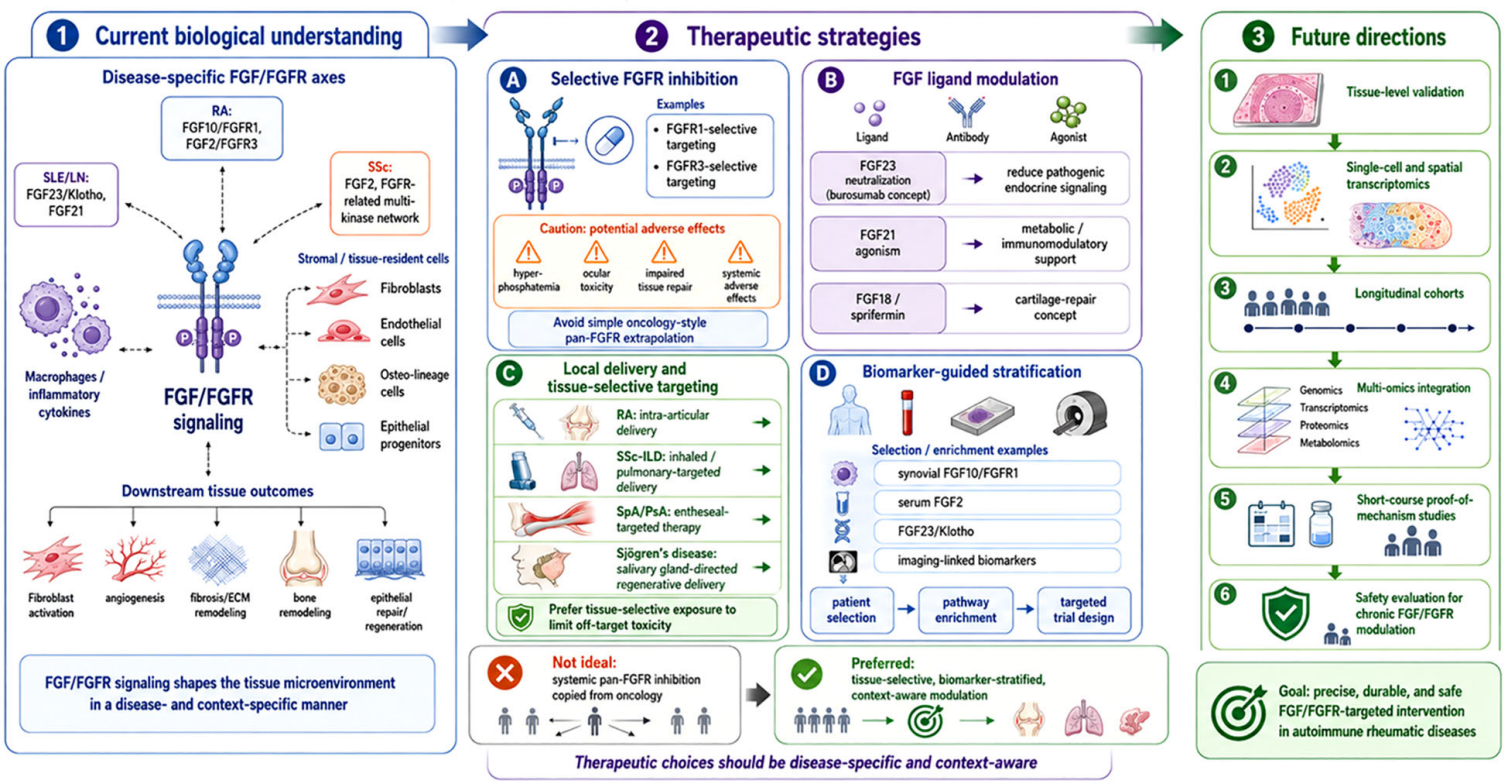
Therapeutic strategies and future roadmap for targeting FGF/FGFR pathways in autoimmune rheumatic diseases. Therapeutic modulation of FGF/FGFR signaling in autoimmune rheumatic diseases requires a strategy distinct from the oncology paradigm of systemic pan-FGFR inhibition. Potential approaches include selective FGFR modulation, FGF ligand modulation, local or tissue-selective delivery, and biomarker-guided patient stratification. Except for nintedanib in SSc-ILD, these approaches remain translational concepts requiring validation rather than clinical recommendations. Ligand-directed strategies, including FGF23 neutralization, FGF21 agonism, and FGF18-based tissue repair, remain largely extrapolated from non-autoimmune contexts and require dedicated validation. Local delivery approaches, such as intra-articular, pulmonary-targeted, entheseal-targeted, or gland-directed administration, may reduce systemic toxicity and preserve homeostatic FGF functions. Future priorities include tissue-level validation, single-cell and spatial mapping, ligand-receptor-isoform specificity, longitudinal biomarker cohorts, short-course proof-of-mechanism trials, and safety-conscious local delivery strategies.

The translational question is not whether FGF/FGFR signaling can be broadly targeted in autoimmune rheumatic diseases, but which axis, in which tissue, in which patient subgroup, and with which pharmacodynamic endpoint should be prioritized. Except for nintedanib in SSc-ILD, no FGF/FGFR-directed strategy has been clinically validated for autoimmune rheumatic diseases. Therefore, this section provides an axis-specific translational prioritization framework rather than therapeutic recommendations. The framework links evidence strength to the appropriate next step: tissue validation for candidate mechanisms, longitudinal prognostic testing for biomarker axes, and short-course proof-of-mechanism studies only for axes with sufficient tissue-level support (**Figure 3**; **Table 4**).

**Table 4:** Axis-prioritization translational roadmap.

Disease	Priority axis	Evidence level	Patient subgroup	Validation tissue/sample	Intervention/delivery concept	PD endpoint	Clinical/imaging endpoint
RA	FGF10/FGFR1	Strong mechanistic	Remission patients at high relapse risk	Synovial biopsy; synovial fluid	Local FGFR1 modulation; short-course proof-of-mechanism	pERK; CD55+ lining FLS; Ki67; FGF10/FGFR1 activity	Flare after tapering; Doppler synovitis; MRI synovitis
RA	FGF2/FGFR3	Strong/moderate mechanistic	Erosive or FLS-dominant RA	Synovium; synovial fluid	FGFR3/RSK2 pathway modulation; local preferred	MMP-1/3/13; RANKL; osteoclast markers	Erosion progression; MRI/US synovitis
SLE/LN	FGF23/Klotho-MCP-1/CCL2 interface	Biomarker-level	Active LN; renal-metabolic inflammatory phenotype	Serum; urine; renal biopsy when available	Biomarker validation, not therapy	FGF23/Klotho; urinary MCP-1/CCL2; tubular/endothelial markers	Renal flare; proteinuria; eGFR; biopsy activity/chronicity
SSc-ILD	FGFR-containing multi-kinase network	Clinical translational, not FGFR-specific	Progressive fibrosing SSc-ILD	Blood; BAL; HRCT; optional tissue	Nintedanib biomarker substudy; selective FGFR comparator in models	FGFR pathway engagement; fibroblast activation markers	FVC decline; HRCT fibrosis progression
SpA/PsA	FGF2 angiogenesis-osteogenesis; FGF7/FGFR2IIIb	Moderate/preliminary	Active enthesitis; early structural progression	Enthesis imaging; serum; tissue if feasible	Validation first; local enthesis targeting only experimental	FGF2/FGF7; vascular and osteogenic markers	MRI enthesitis; syndesmophyte/new bone formation
Sjögren	FGF7/FGF10/FGFR2b	Extrapolated regenerative	Residual glandular tissue/function	Salivary gland biopsy; patient-derived organoids	Local glandular regenerative delivery only after model validation	Epithelial progenitor response; acinar/ductal markers	Salivary flow; glandular imaging; dryness score

### 9.1 FGFR inhibition: opportunities and safety considerations

Several selective FGFR inhibitors have been approved for oncologic indications, including erdafitinib (pan-FGFR1-4, urothelial carcinoma), pemigatinib (FGFR1-3, cholangiocarcinoma), and futibatinib (irreversible pan-FGFR1-4, cholangiocarcinoma) (115, 116). While these agents provide proof that FGFR kinase activity can be pharmacologically suppressed, the oncology paradigm of chronic systemic FGFR blockade is poorly suited to autoimmune rheumatic diseases, where long-term safety, tissue repair, fertility, bone homeostasis, and ocular toxicity must be considered in patients with non-malignant chronic disease.

The safety profile of chronic FGFR inhibition raises serious concerns for long-term use in non-oncologic populations. Class-effect adverse events include hyperphosphatemia (due to blockade of the FGF23/FGFR1c/Klotho axis in the kidney), soft tissue mineralization, nail toxicity, mucositis, impaired tissue repair, and central serous retinopathy/ocular toxicity (115, 117). In oncology, these toxicities may be acceptable in the context of life-threatening disease and finite treatment windows; in autoimmune diseases requiring chronic or repeated therapy, the risk-benefit calculus is fundamentally different.

A more rational approach may involve isoform-selective FGFR targeting. For example, selective FGFR1 inhibition could target the FGF10/FGFR1 relapse axis in RA without affecting FGFR2IIIb-dependent epithelial repair. Similarly, selective FGFR3 inhibition could target the FGF2/FGFR3/RSK2 axis in RA-FLS (23, 57). Experimental anti-FGFR2IIIb strategies developed for oncology could theoretically be repurposed for entheseal bone formation in PsA, though safety data in non-oncologic populations are lacking (52). FGF trap and decoy receptor approaches, which sequester FGF ligands rather than blocking receptor kinase activity, may offer a more nuanced mechanism with potentially fewer systemic side effects (59).

### 9.2 FGF ligand modulation

Rather than blocking FGFR signaling, an alternative strategy involves modulating specific FGF ligands. Three agents illustrate the range of possibilities.

Burosumab, a monoclonal antibody against FGF23, is approved for X-linked hypophosphatemia and illustrates that ligand-level FGF23 neutralization is pharmacologically feasible (118). Its relevance to rheumatic diseases remains uncertain and would require a clearly defined subgroup with pathogenic, not merely compensatory, FGF23 dysregulation. Because FGF23 neutralization can increase phosphate levels and theoretically promote ectopic mineralization, any rheumatic-disease application would require strong mechanistic justification, careful patient selection, and close mineral-metabolism monitoring.

FGF21 analogues (pegbelfermin, efruxifermin, efimosfermin alfa) are in clinical development for non-alcoholic steatohepatitis/metabolic dysfunction-associated steatohepatitis (NASH/MASH) (119–121). Given the preclinical evidence for anti-inflammatory and joint-protective effects of FGF21 in CIA models (31, 32, 66), FGF21 agonism remains a speculative therapeutic concept for RA. No clinical evidence currently supports FGF21 agonism as therapy for human RA or other autoimmune rheumatic diseases. The metabolic benefits of FGF21 may be relevant to autoimmune disease patients with metabolic comorbidities, but this remains separate from evidence for disease-modifying immunological efficacy.

Sprifermin (recombinant human FGF18) has been evaluated for intra-articular cartilage repair in knee osteoarthritis, with the FORWARD trial demonstrating dose-dependent increases in cartilage thickness (53, 122, 123). Sprifermin should be viewed as proof-of-concept for local FGF delivery to joint tissues rather than evidence for FGF-based treatment of inflammatory arthritis. Its mechanism (FGFR3-mediated chondrocyte stimulation) addresses cartilage biology rather than the immune-stromal mechanisms central to RA.

It is important to note that the majority of evidence for these FGF ligand modulators comes from non-autoimmune contexts. Their applicability to rheumatic diseases requires dedicated preclinical and early clinical evaluation.

### 9.3 Tissue-selective delivery and proof-of-mechanism design

We propose that the most promising translational direction for FGF/FGFR targeting in autoimmune diseases is tissue-selective intervention rather than systemic drug administration. Several disease-specific applications can be envisioned:

Intra-articular delivery for RA: the most plausible early test would be local FGFR1 or FGFR3 pathway modulation in patients with relapse-prone or erosive synovitis, using ultrasound-guided synovial biopsy, synovial fluid sampling, or imaging-based synovitis measures to assess target engagement. Pharmacodynamic readouts could include pERK activation, FGF10/FGFR1 or FGF2/FGFR3 pathway activity, CD55+ lining FLS abundance, Ki67, MMP-3, RANKL, osteoclastogenic markers, and Doppler synovitis. The sprifermin experience shows that intra-articular FGF-pathway delivery is technically feasible in joint tissues (53).

For SSc-ILD, translational work should prioritize biomarker-enriched nintedanib substudies and preclinical selective FGFR comparator experiments rather than assuming FGFR causality. For SpA/PsA, local or imaging-linked studies should test whether FGF2 or FGF7 signals track active enthesitis and subsequent new bone formation. For Sjögren’s disease, any FGF7/FGF10-based regenerative approach should first be tested in patient-derived organoids or inflamed gland models before local salivary gland delivery is considered.

In all cases, proof-of-mechanism studies with tissue-level pharmacodynamic endpoints should precede large clinical trials. Short-course interventions with clear mechanistic readouts are more appropriate for this early-stage field than long-duration outcome trials.

### 9.4 Biomarker-stratified validation before therapeutic targeting

Biomarker stratification should be matched to evidence strength. In RA, synovial FGF10/FGFR1 expression at remission may identify patients at risk of relapse after treatment tapering, whereas synovial or synovial-fluid FGF2/FGFR3 activity may enrich for FLS-driven erosive disease. In SLE/LN, serial FGF23/Klotho measurements should be evaluated together with urinary MCP-1/CCL2, proteinuria, eGFR, complement, anti-dsDNA, and renal biopsy activity/chronicity indices to determine whether this axis provides independent prognostic information. In SSc-ILD, FGFR-related biomarkers should be tested as modifiers of nintedanib response rather than as stand-alone treatment-selection markers. In SpA/PsA, serum or tissue FGF2/FGF7 signals should be linked prospectively to MRI enthesitis and radiographic new bone formation. Without such stratification, FGF/FGFR targeting risks repeating the oncology paradigm of pathway inhibition without autoimmune disease-specific rationale.

To make the translational framework explicit, **Table 4** summarizes each candidate FGF/FGFR axis according to disease context, evidence strength, patient subgroup, validation tissue or sample, delivery concept, pharmacodynamic endpoint, and clinical or imaging endpoint. This table is intended to prevent premature therapeutic extrapolation: biomarker-level axes are assigned to longitudinal validation, extrapolated regenerative axes to patient-derived models, and only tissue-supported axes to proof-of-mechanism intervention.

## 10 Discussion: challenges, limitations, and future directions

Despite growing interest in FGF/FGFR signaling, most autoimmune rheumatic disease evidence remains early-stage. Future work should not treat all FGF axes equally. Instead, research priorities should follow the evidence tier: tissue-supported axes require pharmacodynamic validation, biomarker-level axes require longitudinal prognostic testing, and extrapolated regenerative axes require patient-derived disease models. This tiered approach is necessary to avoid overinterpreting preliminary associations as disease mechanisms (**Table 5**).

**Table 5:** Research priorities for FGF/FGFR studies in autoimmune rheumatic diseases.

Priority	Core task	Minimum study design	Critical endpoint	Why it matters
Tissue-level validation	Confirm local ligand/receptor expression in disease tissue	Synovial, renal, skin/lung, entheseal, or glandular tissue profiling	Spatial co-localization with disease-relevant cells	Separates disease mechanisms from peripheral biomarker noise
Single-cell and spatial mapping	Map ligand-receptor interactions by cell type and location	scRNA-seq plus spatial transcriptomics/proteomics	Validated FGF/FGFR cellular niches	Prioritizes axes for functional testing
Ligand-receptor-isoform specificity	Define ligand, receptor isoform, target cell, and disease stage	Isoform-aware assays and functional perturbation	Cell-type-specific pathway response	Avoids broad, misleading pathway generalizations
Longitudinal biomarker cohorts	Test whether FGF/Klotho markers predict outcomes	Serial serum/urine/tissue measures with imaging and clinical follow-up	Independent prognostic value beyond standard markers	Determines biomarker utility rather than association alone
Short-course proof-of-mechanism trials	Use brief interventions with tissue pharmacodynamics	Small biomarker-enriched studies before efficacy trials	Target engagement and pathway modulation	Prevents premature therapeutic claims
Safety and local delivery strategies	Prioritize local or tissue-selective modulation	Preclinical safety plus local delivery feasibility studies	Toxicity, repair, bone, ocular, and metabolic endpoints	Matches risk tolerance for non-malignant chronic disease

### 10.1 Tissue-level validation

The first priority is disease-tissue validation. In RA, ultrasound-guided synovial biopsy and synovial fluid analyses can test whether FGF10/FGFR1 or FGF2/FGFR3 activity is spatially linked to lining FLS, macrophages, endothelial cells, and osteoclastogenic niches. In LN, renal biopsy studies should map FGF23, FGFRs, Klotho, urinary MCP-1/CCL2-related chemokine programs, tubular injury, endothelial activation, and macrophage infiltration. In SSc, paired skin, lung, blood, and imaging data are needed to separate FGFR-related fibrosis from PDGFR/VEGFR effects. In SpA/PsA, direct entheseal tissue is difficult to obtain, so imaging-linked serum and tissue-adjacent models may be required. In Sjögren’s disease, minor salivary gland biopsy and patient-derived organoids should determine whether FGF-responsive epithelial or progenitor populations persist in damaged glands.

### 10.2 Single-cell and spatial mapping

The second priority is systematic single-cell and spatial mapping of ligand-receptor relationships. Integration of single-cell transcriptomics, spatial proteomics, and longitudinal clinical phenotyping may help prioritize disease-relevant FGF/FGFR axes. Computational ligand-receptor inference tools should be used to generate hypotheses, but predicted interactions require experimental validation in disease tissues.

### 10.3 Ligand-receptor-isoform specificity

The third priority is specificity. Studies should distinguish FGF ligand, FGFR isoform (IIIb versus IIIc), target cell type, and disease stage rather than treating “FGF signaling” as a monolithic entity. The dual nature of FGF2 in SSc and the divergent implications of FGF23 as inflammatory mediator versus renal-metabolic marker illustrate why broad pathway labels are insufficient for translational decisions.

### 10.4 Longitudinal biomarker cohorts

The fourth priority is prospective longitudinal biomarker research. Most FGF/Klotho studies in rheumatic diseases remain cross-sectional, single-center, and underpowered. Cohorts with serial FGF/Klotho measurements, conventional disease markers, imaging, tissue sampling where feasible, and prespecified outcomes are needed to determine whether FGF signals add prognostic value or merely track established damage and comorbidity.

### 10.5 Short-course proof-of-mechanism trials

Short-course proof-of-mechanism studies should be considered only for axes with adequate tissue-level support. A possible RA design would enroll patients with active or relapse-prone synovitis, apply a local or short-course pathway-modulating intervention, and measure synovial pERK, FGF-responsive FLS subsets, MMPs, RANKL, osteoclastogenic markers, and imaging synovitis before and after exposure. For SSc-ILD, mechanistic substudies of nintedanib should measure FGFR-pathway engagement alongside VEGFR and PDGFR markers to determine whether any treatment response is FGFR-enriched. For SLE/LN, SpA/PsA, and Sjögren’s disease, the current evidence is insufficient for interventional trials; longitudinal biomarker studies, tissue mapping, and patient-derived models should come first.

### 10.6 Safety and local delivery strategies

The sixth priority is safety-conscious delivery. Chronic systemic FGFR blockade carries risks of hyperphosphatemia, soft tissue mineralization, ocular toxicity, impaired tissue repair, skeletal effects, and reproductive safety concerns (115, 117). Local delivery systems, tissue-selective prodrugs, inhaled pulmonary approaches, and short-course treatment paradigms should be evaluated before systemic FGFR inhibition is considered for non-malignant autoimmune disease.

Overall, the future of this field depends on tissue-specific, cell-type-informed, and biomarker-guided evaluation of selected FGF/FGFR axes rather than broad claims about FGF signaling as a uniform therapeutic target.

### 10.7 Limitations of this review

This review has several limitations. First, it is a narrative, evidence-informed synthesis rather than a systematic review. We did not perform PRISMA-based screening, risk-of-bias assessment, protocol registration, or meta-analysis. Second, the evidence base is highly uneven across diseases. RA and SSc-ILD have stronger tissue-level or clinical translational data, whereas SLE/LN, SpA/PsA, and Sjögren’s disease rely more heavily on biomarker studies, animal models, organoids, or developmental biology extrapolation. Third, many studies measure circulating FGF or Klotho levels, which may reflect renal function, inflammation, cardiovascular risk, age, mineral metabolism, or treatment exposure rather than disease-specific mechanisms. Fourth, ligand-receptor and FGFR isoform specificity is often incompletely defined. Finally, this review proposes a translational prioritization framework, but the framework itself requires prospective validation in disease-tissue and clinical cohorts.

### 10.8 Conclusion

This review synthesizes evidence on FGF/FGFR signaling across five autoimmune rheumatic disease contexts and highlights both the promise and the limitations of this field. Rather than supporting a uniform FGF/FGFR mechanism across diseases, the current literature reveals a heterogeneous landscape of axis-, tissue-, and disease-specific biology.

In RA, FGF10/FGFR1 and FGF2/FGFR3 have the strongest mechanistic support, linking synovial fibroblast niches, FLS activation, angiogenesis, and bone erosion. In SLE/LN, FGF23/Klotho is best viewed as an emerging renal-metabolic and inflammatory biomarker axis; its association with urinary MCP-1/CCL2 provides a mechanistic clue but not yet proof of causality. In SSc-ILD, nintedanib shows that FGFR-containing multi-kinase networks are clinically tractable, while the FGFR-specific contribution remains unresolved. In SpA/PsA, FGF2 and FGF7/FGFR2IIIb provide candidate mechanisms for entheseal remodeling but require human validation. In Sjögren’s disease, FGF7/FGF10/FGFR2b signaling supports a regenerative hypothesis that must be tested in autoimmune-damaged glands and patient-derived models (**Figures 2**, **3**).

Three principles follow from this synthesis. First, FGF/FGFR signaling should be understood as part of a broader tissue microenvironment remodeling network that interacts with inflammatory cytokines, chemokines such as MCP-1/CCL2, extracellular matrix proteins, and mechanical cues. Second, therapeutic translation should not follow the oncology model of chronic systemic pan-FGFR inhibition; it should prioritize tissue-selective, locally delivered, biomarker-stratified, and safety-conscious approaches. Third, the field must move from peripheral biomarker association toward tissue-level validation, ligand-receptor-isoform specificity, longitudinal cohorts, and short-course proof-of-mechanism studies. Selected FGF/FGFR axes may eventually become useful biomarkers or therapeutic entry points, but their clinical relevance will depend on rigorous validation matched to evidence strength.
